# Neural measures of the role of affective prosody in empathy for pain

**DOI:** 10.1038/s41598-017-18552-y

**Published:** 2018-01-10

**Authors:** Federica Meconi, Mattia Doro, Arianna Schiano Lomoriello, Giulia Mastrella, Paola Sessa

**Affiliations:** Department of Developmental and Social Psychology, University of Padova, Padova, Italy

## Abstract

Emotional communication often needs the integration of affective prosodic and semantic components from speech and the speaker’s facial expression. Affective prosody may have a special role by virtue of its dual-nature; pre-verbal on one side and accompanying semantic content on the other. This consideration led us to hypothesize that it could act transversely, encompassing a wide temporal window involving the processing of facial expressions and semantic content expressed by the speaker. This would allow powerful communication in contexts of potential urgency such as witnessing the speaker’s physical pain. Seventeen participants were shown with faces preceded by verbal reports of pain. Facial expressions, intelligibility of the semantic content of the report (i.e., participants’ mother tongue vs. fictional language) and the affective prosody of the report (neutral vs. painful) were manipulated. We monitored event-related potentials (ERPs) time-locked to the onset of the faces as a function of semantic content intelligibility and affective prosody of the verbal reports. We found that affective prosody may interact with facial expressions and semantic content in two successive temporal windows, supporting its role as a transverse communication cue.

## Introduction

In face-to-face interactions, communication has a multi-modal nature involving the processing of visual facial cues (such as the speaker’s facial expression), the tone of the voice (i.e., affective prosody) and the choice of words (i.e., semantics^1–6^).

In the current event-related potential (ERP) study we provided evidence that when empathizing with others’ pain affective prosody of the speech may interact with both the speaker’s facial expression and the expressed linguistic content (i.e., semantics) in two successive temporal windows. This characteristic can facilitate the understanding of the communication of potential urgency, such as when the speaker expresses physical pain by their facial expression and/or tone of the voice, or when semantic content (i.e., the words of their verbal reports) is not accessible. Indeed, multi-modal communication can improve detection and comprehension of others’ emotions and affective states.

When witnessing others’ physical pain, empathy is often triggered in the observer. Empathy is the ability to share others’ emotional experiences (experience-sharing) and to explicitly infer others’ inner states (mentalizing)^7^; see also^8,9^. At the neuroanatomical level, the two aspects of empathy are dissociable with “experience-sharing” mechanisms engaging the mirror neurons and the limbic systems and “mentalizing” engaging regions of the prefrontal and temporal cortices and precuneus^10–14^. In their influential review on empathy, Zaki and Ochsner^7^ suggested that “although neuroimaging can distinguish the spatial profiles of neural systems associated with experience sharing and mentalizing, electrophysiological techniques are more useful for elucidating the temporal dynamics of these processes”. There is, indeed, evidence that this neuroanatomical distinction is revealed also at the functional level as revealed by electrophysiological studies^15,16^. In this vein, Zaki and Ochsner cite an ERP study – now considered one of the earliest in the field – that elegantly revealed two successive temporal windows of neural activity reflecting experience sharing and mentalizing, respectively^15^. The authors administered participants with a classic version of the pain decision task and presented participants with one or two hands in neutral or in painful conditions. Participants were required to indicate whether the hands were depicted in either the painful or neutral conditions (i.e., pain decision task) or to indicate whether one or two hands were presented on the screen (i.e., counting task). The authors observed modulations in amplitude related to the processing of the painful condition of early (N1, P2, and N2–N3) and late components (P3) in the pain decision task manifest as a positive shift of the painful condition when compared to the neutral condition. Crucially, when attentional resources were withdrawn from the painful information (i.e., in the counting task) the later P3 response was reduced to nil suggesting that the earlier and the later responses reflected more automatic (versus controlled) mechanisms of empathy.

In line with this evidence, experience-sharing and mentalizing can be selectively activated depending on the nature of the available cue, perceptual and non-perceptual, respectively^17^, see also^18^. In a more recent ERP study, Sessa *et al*.^16^ supported the view that the nature of information available to the observers is crucial in order to selectively trigger experience-sharing (i.e., empathic reactions to painful facial expressions triggering P2 and N2–N3 ERP modulations) or rather mentalizing (i.e., empathic reactions to verbal information of pain modulating the later P3 ERP component). Previous studies, both in the contexts of empathy^19^ and recognition of emotional faces^20^ have observed very similar ERP modulations elicited by facial expressions. In our previous studies we estimated the neural sources of the early and late ERP modulations^16,21^ and found evidence compatible with previous work supporting two anatomically and functionally dissociable brain networks underlying experience sharing and mentalizing processes, respectively^7^. Moreover, we observed that these modulations correlated with explicit measure of dispositional empathy. That is, the N2–N3 ERP reaction to pain was significantly correlated with one of the affective empathy subscales of the Interpersonal Reactivity Index^21^ (IRI^22^, i.e., the Empathic Concern), while the pain effects observed on the P3 component were significantly correlated with one of the cognitive empathy subscales of the IRI^23^ (i.e., the Perspective Taking) and with the Empathy Quotient^24^ (EQ^25^).

Therefore, based on this broad convergence of evidence, researchers in the field interpret the modulations of the above-mentioned ERP components (i.e., positive shift of the ERPs elicited in the painful condition when compared to the neutral condition) as a correlate of empathic processes, underpinning experience sharing and mentalizing processes of empathy, respectively. The evidence reported above strongly supports the notion that these empathic processes can then be triggered by the kind of available cue. Within this body of research, empathic response to pain has been triggered either by facial expression of pain or by body parts undergoing a painful stimulation (e.g., a needle pricking the skin). Other experimental manipulations included written sentences describing painful contexts. In real life, one common way to express pain is through verbal reports. However, that has been less extensively addressed. Such reports include the characteristics of the speaker’s voice expressing pain, such as prosody. The prosodic information clarifies the meaning, the intentions or the emotional content of the speech. The potential impact of prosody and its possible interactions with both facial expression and linguistic content within the two different systems and temporal windows associated with empathic processes is the focus of the current study. As we clarify below, we believe this is particularly relevant to test the framework of empathy that proposes two dissociable systems. Notably, prosody seems to have a dual-nature, it is “pre-verbal” (it is defined by a mix of perceptual characteristics, mainly auditory) but also accompanies language and semantic content^26^.

Previous studies hold the view that prosody can interact with both facial expressions and verbal information. Cross-modal integration of audiovisual emotional signals appears to occur rapidly and automatically with^27,28^ and without conscious awareness^29^. Paulmann *et al*.^27^ used eye-tracker technique to study how prosodic information of instructions delivered trial by trial (e.g., “Click on the happy face”) influenced eye movements to emotional faces within a visual array. Importantly, affective prosody could be either congruent or incongruent with the emotional category of the face to be clicked on the basis of the instructions (e.g., “Click on the happy face” pronounced with a congruent happy prosody or with an incongruent sad, angry or frightened prosody). Participants’ eye movements were monitored before and after the adjective included in the instructions was pronounced. The authors observed longer, frequent fixations to faces expressing congruent emotion than when expressing incongruent emotion with prosodic information. However, the influence of prosody on eye gaze decreased once the semantic emotional information (i.e., the adjective) was presented. In sum, these findings demonstrated that prosodic cues are extracted rapidly and automatically to guide eye gaze on facial features to process facial expressions of emotions. However, the effect of the prosodic cue weakens as the semantic information is unveiled supporting those results showing that even irrelevant semantics cannot be ignored when participants have to discriminate affective prosody of matching or mismatching utterances^30^.

Neuroimaging studies showed rightward lateralization of prosodic processing^30^, in line with brain lesions studies showing that dysprosody, but not aphasia, follows right brain injuries^31,32^; but see also^26^. The idea that semantic and prosodic processing are anatomically and functionally dissociable is not surprising since processing of affective prosodic information appears to be at least in part a pre-verbal ability that can be observed as early as in 7 months-old infants^33,34^ and it is also phylogenetically ancient, as it is present in macaque monkeys^35^.

A recent series of studies by Regenbogen and colleagues used skin conductance response and functional magnetic resonance imaging (fMRI) to investigate the integration of affective processing in multimodal emotion communication^36,37^. Regenbogen and colleagues^36^ exposed participants to video-clips showing actors expressing emotions through a full or partial combination of audio-visual cues such as prosody, facial expression and semantic content of the speech. Their findings showed that the empathic physiological response was limited in the partial (emotion was not expressed by one of the audio-visual cues) when compared to full combination of cues. Convergent evidence with these findings was provided by the authors in a similar neuroimaging study, that further revealed that the neural activation in the full and partial combination of audio-visual cues was very similar, involving brain areas of the mentalizing system^37^ (i.e., lateral and medial prefrontal cortices, orbitofrontal cortex and middle temporal lobe). However, this noteworthy study could not provide a full picture of which components of empathy are influenced by affective prosody nor could it trace the time-course of such influence. In the present study, by means of ERPs, we tried to draw such a picture, and we did so within the theoretical framework of empathy for others’ pain^7,16^.

In the current study, we monitored neural empathic responses towards individuals expressing physical pain through verbal reports of painful experiences followed by facial expressions. ERP responses were time-locked to facial expressions. We orthogonally manipulated “facial expressions” (neutral vs. painful), the semantic accessibility of the verbal reports expressing pain (i.e., utterances in mother-tongue vs. utterances in a fictional language designed to sound natural; we named this manipulation “intelligibility”: intelligible vs. unintelligible utterances) and the “prosody” of the verbal reports (neutral vs. painful). To note, the content of intelligible utterances was always of pain. The two sets of utterances (in mother-tongue and in fictional language) were declaimed by a professional actor so that the prosody of each utterance matched between languages. An independent sample of participants judged the intensity of pain conveyed by the prosody of each utterance confirming that the perception of the pain expressed by the tone of the voice did not differ between the two sets of utterances.

We also collected explicit measures of participants’ dispositional empathy (i.e. Empathy Quotient^25^ and Interpersonal Reactivity Index^22^). In line with our previous findings^16^, we expected painful facial expressions and intelligible utterances (always with a content of pain) to trigger dissociable empathic reactions in two successive temporal windows. We time-locked ERP analysis to the presentation of facial stimuli as a function of preceding utterances and we anticipated that facial expressions would have selectively elicited empathic reactions on the P2 and N2–N3 ERP. Lastly, we expected intelligible utterances (i.e., utterances expressing a painful context in participants’ mother-tongue) to trigger empathic reactions on the P3 ERP component when compared to unintelligible utterances (i.e., in a fictional language). On the basis of previous studies we expected these empathic reactions to manifest as positive shifts of ERPs time-locked to faces onset for painful facial expressions and intelligible utterances when compared to neutral conditions^15,16,19,21,24^. The current study was specifically designed to unravel the role of affective prosody in inducing an empathic response as an additional cue of others’ pain. We hypothesized and demonstrated that, by virtue of its dual-nature, affective prosody can be considered cross-domain information able to transversely influence processing of painful cues triggering experience-sharing (facial expressions; *pre-verbal*) and mentalizing responses (intelligible utterances with a content of pain; *verbal domain of processing*). More specifically, we anticipated that affective prosody would have affected the neural empathic response to painful facial expressions in the early temporal window linked to experience-sharing (i.e., P2, N2–N3 ERP reaction, time-locked to faces onset), and the empathic response to painful intelligible utterances in a dissociable and later temporal window associated with mentalizing (i.e., P3 reaction, always time-locked to the onset of faces).

## Results

### Questionnaires

The present sample of participants showed a mean EQ score in the middle empathy range according to the original study^25^, i.e. 46.83 (*SD* = 7.09). IRI scores were computed by averaging the scores of the items composing each subscale as reported in Table 1.

**Table 1:** IRI scores.

IRI			
Cognitive		Affective	
*Pt*	3.69 (0.41)	*EC*	3.83 (0.53)
*F*	3.55 (0.66)	*PD*	2.66 (0.66)

### Behavior

Participants were more accurate when prosody of the reports and the facial expression of the faces were congruent, as indexed by the interaction between the factors prosody and facial expression, *F*(1,16) = 6.086, *p* = 0.025, *MS*_*e*_ = 0.000184, *η*_*p*_^2^ = 0.276 – independently of the intelligibility of the semantic content – and post-hoc t-test (*t(16)* = 2.467, *p* = 0.025, *M*_*diff*_ = 0.007 [0.002, 0.014]). No main effect or other interactions between factors reached significance level (max *F* = 3.794, min *p* = 0.069, max *η*_*p*_^2^ = 0.192). An ANOVA did not show any significant result for RTs (max *F* = 3.570, min *p* = 0.077, max *η*_*p*_^2^ = 0.182).

**Figure 1:**
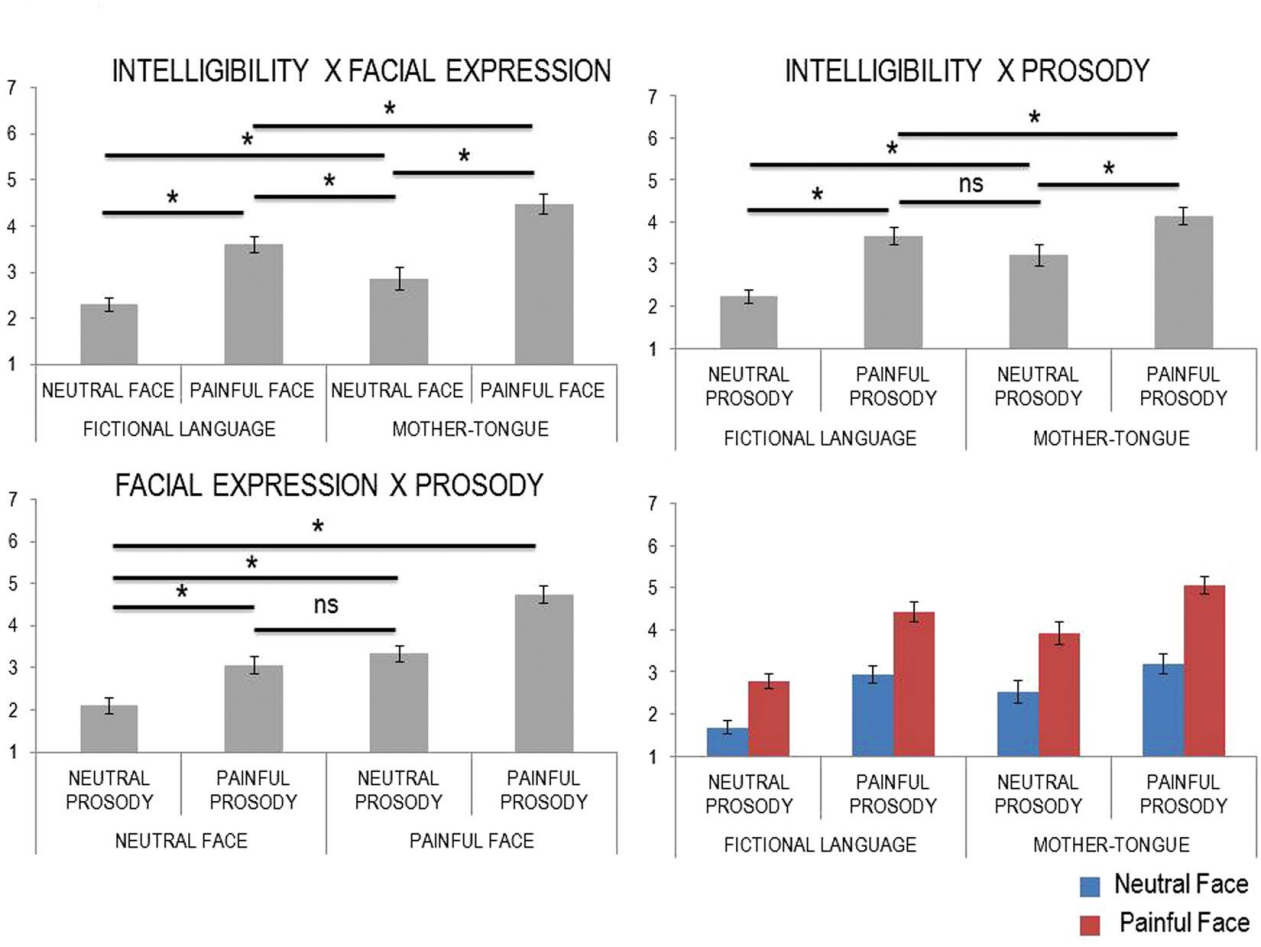
Rating results showing significant and non-significant comparisons for the interactions (upper panel and bottom left panel) and the whole pattern of results (bottom right panel). Error bars represent standard errors, asterisks represent significant comparisons; “n.s.” means “not-significant”.

An ANOVA on individual rating scores showed the main effects of facial expression, *F*(1,16) = 126.405, *p* < 0.000001, *MS*_*e*_ = 0.575, *η*_*p*_^2^ = 0.888, intelligibility, *F*(1,16) = 25.063, *p* = 0.000129, *MS*_*e*_ = 0.714, *η*_*p*_^2^ = 0.610, and prosody, *F*(1,16) = 55.270, *p* = 0.000001, *MS*_*e*_ = 0.863, *η*_*p*_^2^ = 0.776. All cues induced higher scores of self-rated empathy in painful conditions relative to neutral conditions. The two-way interaction between facial expression and prosody was significant, *F*(1,16) = 10.219, *p* = 0.006, *MS*_*e*_ = 0.229, *η*_*p*_^2^ = 0.390. Post-hoc t-tests revealed that participants rated their empathy as higher when both facial expression and prosody were painful compared to neutral, (*t(16)* = 10.526, *p* < 0.000001). Both conditions in which only one of the cues was painful induced significantly higher scores than the condition in which both cues were neutral (min *t(16)* = 6.468, max *p* = 0.000008), but scores did not differ between these conditions when only one cue was painful (*t(16)* = 1.905, *p* = 0.075). The two-way interaction between intelligibility and prosody (*F*(1,16) = 10.219, *p* = 0.006, *MS*_*e*_ = 0.229, *η*_*p*_^2^ = 0.390) indicated that the difference in the rates assigned to painful and neutral prosody was higher when utterances were in a fictional language, when compared to those in participants’ mother-tongue, (*t(16)* = 3.197, *p* = 0.006; *M*_*diff*_ = 0.524 [0.177, 0.872]). Empathy for unintelligible utterances reported with both neutral and painful prosody were rated as lower than intelligible utterances pronounced with neutral and painful prosody (min *t(16)* = 3.252, max *p* = 0.005). Rates to intelligible utterances with neutral prosody did not significantly differ from unintelligible utterances with painful prosody (*t(16)* = 1.945, *p* = 0.07). This pattern could be due to the explicit painful context expressed by intelligible utterances despite being pronounced with neutral prosody. Both unintelligible and intelligible utterances pronounced with painful prosody were rated as higher than those pronounced with neutral prosody (min *t(16)* = 6.320, max *p* = 0.00001). The two-way interaction between facial expression and intelligibility, (*F*(1,16) = 5.135, *p* = 0.038, *MS*_*e*_ = 0.183, *η*_*p*_^2^ = 0.243), revealed that painful, relative to neutral, faces induced higher self-rated empathy following utterances in participants’ mother-tongue compared to those in a fictional language, (*t(16)* = −2.266, *p* = 0.038; *M*_*diff*_ = −0.332 [−0.643, −0.021]), indexing an enhanced self-perceived empathy when both semantic and facial information conveyed pain. All the possible comparisons were significant (min *t(16)* = 3.493, max *p* = 0.003). The three-way interaction did not approach significance (*F* < 1). Figure 1 summarizes the whole pattern of results.

#### ERPs

Preliminary repeated measures ANOVA was carried out with the following factors within-subjects: component (P2 vs. N2–N3 vs. P3), area (fronto-central, *FC*, vs. centro-parietal, *CP*), hemisphere (left vs. right), facial expression (neutral vs. painful), intelligibility (intelligible vs. unintelligible utterance) and prosody (neutral vs. painful).

**Figure 2:**
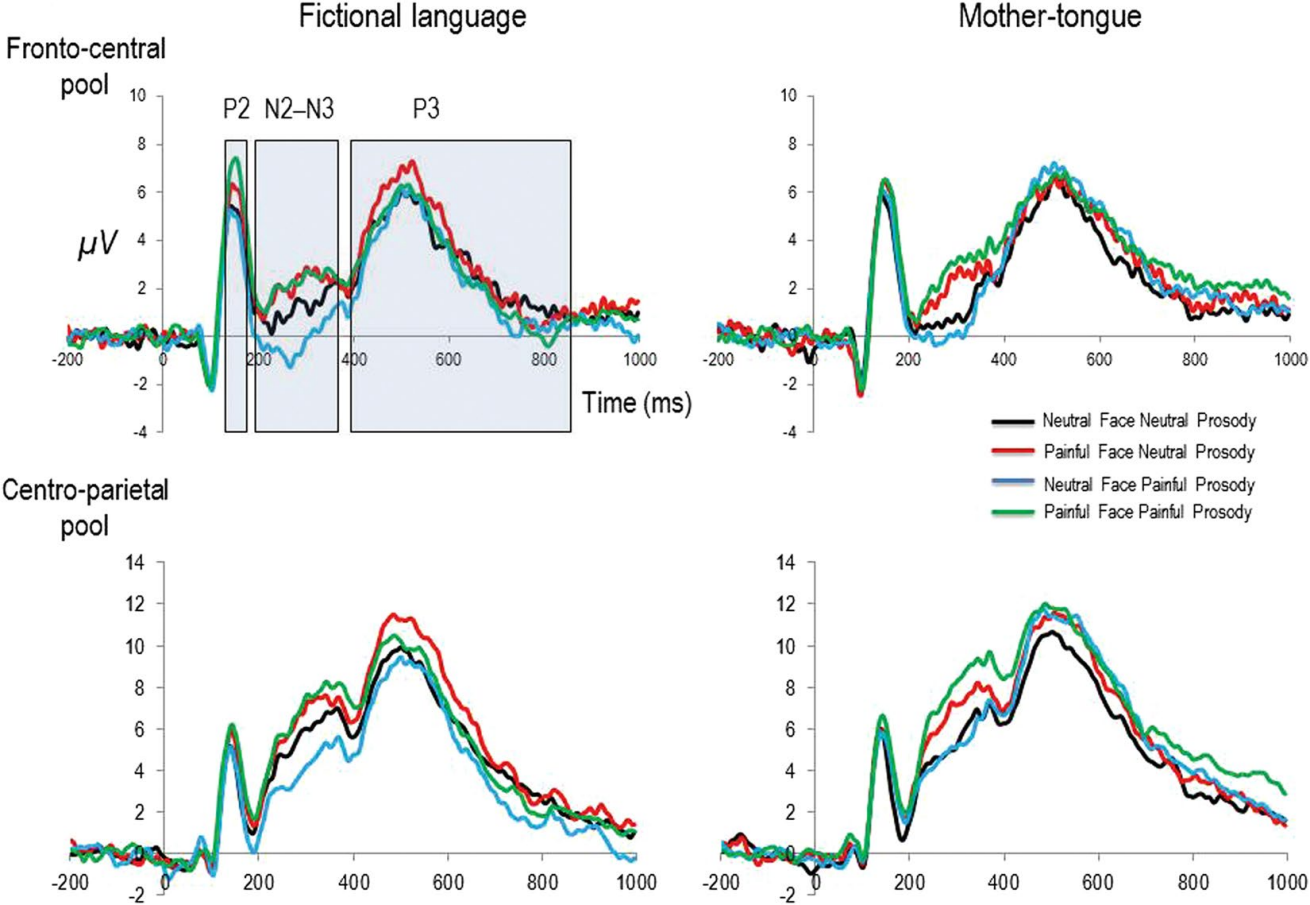
Grand averages of ERPs time-locked to the onset of faces recorded at *FC* (i.e., pooled rFC and lFC), and at *CP* (i.e., pooled rCP and lCP), as a function of preceding utterances superimposed with ERPs elicited in the neutral condition (i.e., neutral prosody/neutral facial expression) separately for participants’ mother-tongue and fictional language.

We observed a main effect of component, *F*(1,16) = 4.036, *p* = 0.04, *η*_*p*_^2^ = 0.350; of area, *F*(1,16) = 13.068, *p* = 0.02, *η*_*p*_^2^ = 0.450; a main effect of intelligibility, *F*(1,16) = 20.765, *p* = 0.0003, *η*_*p*_^2^ = 0.565; and of facial expression, *F*(1,16) = 20.315, *p* = 0.0004, *η*_*p*_^2^ = 0.559. Importantly, we observed significant interaction between component and area, *F*(2,15) = 16.839, *p* = 0.0001, *η*_*p*_^2^ = 0.692; the interaction between component and facial expression, *F*(2,15) = 7.098, *p* = 0.007, *η*_*p*_^2^ = 0.486, and between component and intelligibility, *F*(2,15) = 8.298, *p* = 0.004, *η*_*p*_^2^ = 0.525. Lastly, we observed a significant interaction between component, facial expression and prosody *F*(2,15) = 8.130, *p* = 0.004, *η*_*p*_^2^ = 0.520. Based on these preliminary interactions with the factors component and area, we carried out a second repeated measures ANOVAs separately for each component, again including area as a within-subjects factor. We observed a significant interaction between the area of the scalp and intelligibility on the P2 component (*F*(1,16) = 6.015, *p* = 0.026, *η*_*p*_^2^ = 0.273). The factor area significantly interacted with intelligibility and prosody (*F*(1,16) = 5.069, *p* = 0.039, *η*_*p*_^2^ = 0.241) and with facial expression (*F*(1,16) = 4.482, *p* = 0.05, *η*_*p*_^2^ = 0.219) on the P3 component. We then conducted separated repeated measures ANOVAs for each area of the scalp on the P2 and on the P3 but not on the N2–N3components (Fig. 2).

### P2

With this component, we expected to observe a main effect of the facial expression. The ANOVA revealed a significant main effect of facial expression irrespective of the hemisphere at both pools, *F*(1,16) = 12.711, *p* = 0.003, *MS*_*e*_ = 3.271, *η*_*p*_^2^ = 0.443 at *FC*, *F*(1,16) = 7.908, *p* = 0.013, *MS*_*e*_ = 5.445, *η*_*p*_^2^ = 0.331 at *CP*: painful facial expressions elicited larger P2 (*FC*: 5.630 μV, *SE* = 0.635; *CP*: 5.260 μV, *SE* = 0.568) than neutral facial expressions (*FC*: 4.848 μV, *SE* = 0.495, *CP*: 4.464 μV, *SE* = 0.690).

### The effect of prosody

The three-way interaction between facial expression, prosody and intelligibility reached significance threshold at *FC*, *F*(1,16) = 4.606, *p* = 0.048, *MS*_*e*_ = 1.680, *η*_*p*_^2^ = 0.224). To highlight the effect of prosody, we conducted separate ANOVAs for neutral and painful prosody with facial expression and intelligibility as within-subject factors. ANOVA conducted for neutral prosody did not reveal any significant effect (max *F*(1,16) = 2.384, min *p* = 0.142, max *η*_*p*_^2^ = 0.130). By contrast, ANOVA conducted for painful prosody revealed a main effect of facial expression, *F*(1,16) = 10.183, *p* = 0.006, *MS*_*e*_ = 1.797, *η*_*p*_^2^ = 0.389 and the interaction between facial expression and intelligibility, *F*(1,16) = 8.066, *p* = 0.012, *MS*_*e*_ = 1.120, *η*_*p*_^2^ = 0.335. Bonferroni corrected post-hoc comparisons revealed that painful faces elicited larger P2 than neutral faces when preceded by utterances in a fictional language, (*t(16)* = 4.033, *p* = 0.001; *M*_*diff*_ = 1.766 [0.84, 2.7]) but not when preceded by intelligible utterances, (*t* < 1).

At CP, we observed a main effect of intelligibility, *F*(1,16) = 7.028, *p* = 0.017, *MS*_*e*_ = 3.110, *η*_*p*_^2^ = 0.305, i.e. larger P2 for utterances in mother-tongue than those in a fictional language, that was further qualified by a three-way interaction between intelligibility, prosody and hemisphere *F*(1,16) = 5.224, *p* = 0.036, *MS*_*e*_ = 0.202, *η*_*p*_^2^ = 0.246. Again, to highlight the effect of prosody, we conducted separate ANOVAs for neutral and painful prosody with hemisphere and intelligibility as within-subject factors. ANOVA conducted for neutral prosody, revealed a significant interaction between hemisphere and language, *F*(1,16) = 5.704, *p* = 0.030, *MS*_*e*_ = 0.107, *η*_*p*_^2^ = 0.263. Bonferroni corrected post-hoc comparisons did not reveal any significant effect (max *t(16)* = 1.78, min *p* = 0.094). The ANOVA conducted for painful prosody revealed a main effect of language, *F*(1,16) = 6.232, *p* = 0.024, *MS*_*e*_ = 1.204, *η*_*p*_^2^ = 0.280, showing that intelligible utterances elicited larger P2 than unintelligible utterances.

The main effects of prosody and of hemisphere were not significant, neither were remaining interactions (max *F*(1,16) = 2.688, min *p* = 0.121, max *η*_*p*_^2^ = 0.144).

### N2–N3

Based on previous findings, we mainly we expected to observe a main effect of the facial expression manifest as a positive shift of painful when compared to neutral facial expression.

The ANOVA conducted with the factor area as within-subjects factor revealed a main effect of the area *F*(1,16) = 18.862, *p* = 0.001, *MS*_*e*_ = 103.73, *η*_*p*_^2^ = 0.541 and of facial expression *F*(1,16) = 36.588, *p* = 0.000017, *MS*_*e*_ = 9.313, *η*_*p*_^2^ = 0.696. N2–N3 was significantly more negative at FC when compared to that distributed at CP; more importantly, painful facial expression elicited more positive N2–N3 than neutral expression, i.e. an empathic reaction towards painful faces. This effect was more prominent in the right hemisphere as indexed by the interaction between facial expression and hemisphere *F*(1,16) = 6.842, *p* = 0.019, *MS*_*e*_ = 0.421, *η*_*p*_^2^ = 0.300.

### The effect of prosody

The interaction between facial expression and prosody was significant, *F*(1,16) = 7.574, *p* = 0.014, *MS*_*e*_ = 7.516, *η*_*p*_^2^ = 0.321. Planned comparisons revealed that neutral facial expressions preceded by incongruent painful prosody decreased N2–N3 empathic reaction when compared to neutral condition, i.e. neutral faces preceded by congruent neutral prosody, *t(16)* = −3.207, *p* = 0.008; *M*_*diff*_ = −0.821 [−1.39 −0.246]. This indexed larger negativity for neutral faces preceded by painful relative to neutral prosody. By contrast, painful facial expression preceded by congruent painful prosody increased N2–N3 empathic reaction when compared to the neutral condition, *t(16)* = 3.608, *p* = 0.002; *M*_*diff*_ = 1.41 [−3.608 −0.582]. This empathic reaction was not enhanced when compared to the empathic reaction to painful facial expression preceded by neutral prosody, *t(16)* = 1.383, *p* = 0.186; *M*_*diff*_ = 0.473 [−0.252 1.198]. Remarkably, painful facial expressions preceded by neutral prosody did elicit an N2–N3 empathic reaction relative to the neutral condition, *t(16)* = 2.655, *p* = 0.017; *M*_*diff*_ = 0.936 [0.188 1.683].

No main effect of intelligibility *F*(1,16) = 3.567, *p* = 0.077, *MS*_*e*_ = 4.405, *η*_*p*_^2^ = 0.182 nor of prosody or hemisphere were observed (both *Fs* < 1). None of the other two-way, three-way and four-way interactions were significant (max *F*(1,16) = 4.076, min *p* = 0.061, max *η*_*p*_^2^ = 0.203).

**Figure 3:**
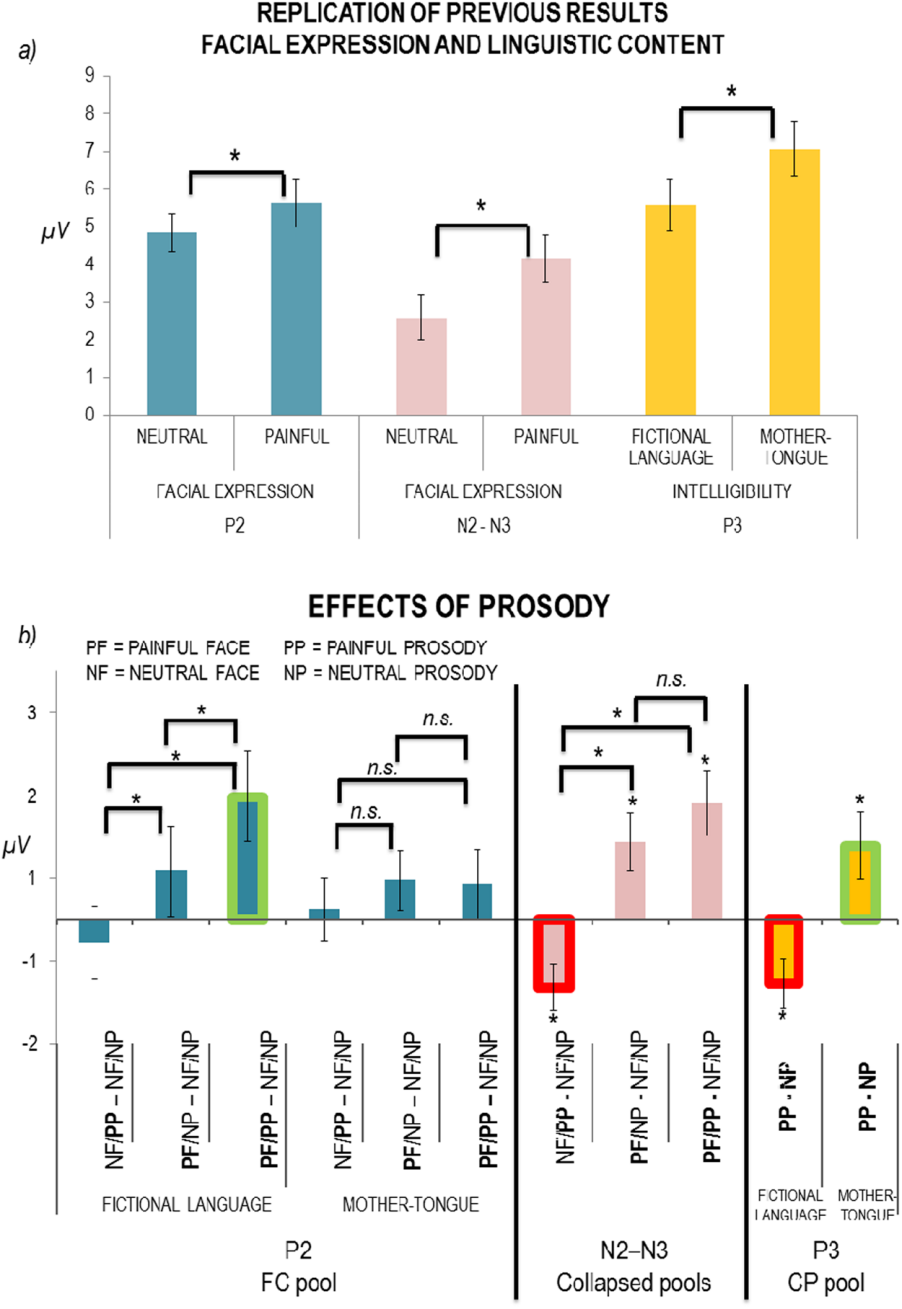
(**a**) Bar graphs showing main effects of facial expression on the P2 and on the N2–N3 components and of the intelligibility on the P3 component. (**b**) Bar graphs showing the effect of prosody on empathic reactions for each ERP component. Empathic reactions are shown as the difference between painful and neutral conditions. Error bars represent standard errors, asterisks significant comparisons, “n.s.” means “not-significant”.

See Fig. 3a for bar graphs representing the main effects of facial expression on the P2 and on the N2–N3 (left and middle panel).

### P3

**Figure 4:**
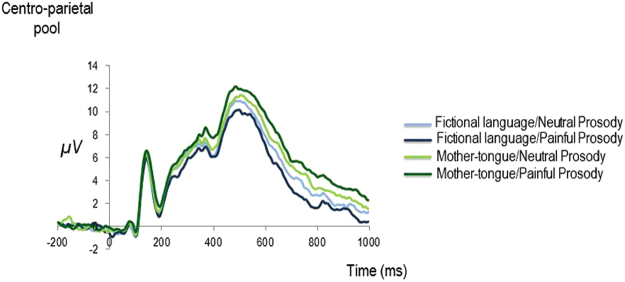
Grand-Averages of ERP time-locked to the onset of faces as a function of language and prosody of preceding utterances recorded at *CP*.

Replicating previous findings, on this component we expected to observe a main effect of the context. In the current study, that was given by the contrast between utterances in mother-tongue, i.e. the context was always painful, and those in a fictional language, where there was no semantic access to the context (Fig. 4).

The ANOVAs revealed a main effect of intelligibility at both sites (*F*(1,16) = 9.143, *p* = 0.008, *MS*_*e*_ = 6.941, *η*_*p*_^2^ = 0.364, at *FC*; *F(1,16)* = 27.477, *p* = 0.000081, *MS*_*e*_ = 5.577, *η*_*p*_^2^ = 0.632, at *CP*) replicating our previous results^16^. P3 time-locked on the onset of the face was larger when faces were preceded by intelligible utterances, i.e. utterances in participants’ mother-tongue, when compared to unintelligible utterances, i.e. in a fictional language.

### The effect of prosody

The interaction between intelligibility and prosody was significant at *CP*, *F*(1,16) = 10.517, *p* = 0.005, *MS*_*e*_ = 4.444, *η*_*p*_^2^ = 0.397 (the same effect was only marginally significant at *FC*, *F*(1,16) = 4.025, *p* = 0.062, *MS*_*e*_ = 4.889, *η*_*p*_^2^ = 0.201). Planned comparisons at *CP* revealed that when content was intelligible, P3 time-locked to the onset of faces did show an empathic reaction to painful prosody, i.e. larger for intelligible utterances pronounced with painful than neutral prosody, *t(16)* = 2.193 *p* = 0.043 (*M*_*diff*_ = 0.89 [0.03 1.74]). When content was unintelligible, such a pattern was not observed: P3 for unintelligible utterances pronounced with painful prosody decreased relative to neutral prosody, *t(16)* = −2.570, *p* = 0.021 (*M*_*diff*_ = −0.77 [−1.40 −0.13]).

We also observed an unexpected modulation of the P3 component due to the facial expression at *CP*, *F*(1,16) = 5.409, *p* = 0.034, *MS*_*e*_ = 6.881, *η*_*p*_^2^ = 0.253 and at rFC as revealed by the significant interaction between hemisphere and facial expression at *FC*, *F*(1,16) = 6.087, *p* = 0.025, *MS*_*e*_ = 1.543, *η*_*p*_^2^ = 0.276 and post-hoc comparisons (*t(16)* = 2.328 *p* = 0.033, *M*_*diff*_ = 0.66 [0.06 1.27] at rFC but not at lFC, *t* < 1): painful facial expressions elicited larger P3 than neutral facial expressions.

Finally, we observed a new significant interaction between hemisphere, facial expression and prosody at *CP*, *F*(1, 16) = 5.613, *p* = 0.031, *MS*_*e*_ = 0.215, *η*_*p*_^2^ = 0.260. To highlight the effect of prosody, we conducted separate ANOVAs for neutral and painful prosody with hemisphere and facial expression as within-subject factors. None of them revealed any significant result, (max *F*(1,16) = 4.045, min *p* = 0.061, max *η*_*p*_^2^ = 0.202.

The main effect of prosody and the other interactions did not reach significance level (max *F*(1,16) = 3.319, min *p* = 0.087, max *η*_*p*_^2^ = 0.172).

See Fig. 3a for bar graph representing the effect of intelligibility on the P3 (right panel). See Fig. 3b for bar graphs representing the effect of prosody on empathic reactions for each time-window. Table 2 summarizes the main results.

**Table 2:** Summary of the main results.

		P2	N2-N3	P3
**Main effects**	***Facial Expression***	Yes - at both FC and CP pools and at both hemispheres, max *p* = 0.013, *η*_*p*_^2^ = 0.331. **Painful facial expressions elicited more positive P2 than neutral expressions**.	Yes, *p* = 0.000017, *η*_*p*_^2^ = 0.696. **Painful facial expressions elicited more positive N2–N3 than neutral expressions.**	*Yes - confined to the CP pool, p* = 0.034, *η*_*p*_^2^ = 0.253, *and to the right FC pool* (see [1] and [3] in “Interactions” row). *Unexpected result: *painful facial expressions elicited larger P3 than neutral faces*.
	***Intelligibility***	Yes - confined to the CP pool, max *p* = *0.017*, η_*p*_^2^ = *0.305.* Larger P2 for utterances in mother-tongue than those in a fictionallanguage. Further qualified by the three-way interaction (see “Interactions” row).	No, *p* > 0.05	Yes - at both FC and CP pools, max *p* = 0.008, *η*_*p*_^2^ = 0.364. **Intelligible utterances elicited larger P3 than utterances in a fictional language**. Further qualified by the two-way interaction (see [2] “Interactions” row).
	***Prosody***	No, *p* > 0.05	No, *p* > 0.05	No, *p* > 0.05
**Interactions**		[1] At FC: **Facial expression x Intelligibility x Prosody,** max *p* = 0.048, *η*_*p*_^2^ = 0.224. Further qualified by separate ANOVA for each level of Prosody (see [1] in the bottom panel). [2] At CP: **Intelligibility x Prosody x Hemisphere** *p* = 0.030, *η*_*p*_^2^ = 0.263.	**Facial expression x Prosody,** *p* = 0.014, *η*_*p*_^2^ = 0.321. Further qualified by post-hoc comparisons (see [3] in the bottom panel).	[1] At FC: **Hemisphere x Facial expression,** *p* = 0.025, *η*_p_^2^ = 0.276. Further qualified by post-hoc comparisons (see [3] in the bottom panel).[2] At CP: **Intelligibility x Prosody,** max *p* = 0.005, *η*_*p*_^2^ = 0.397. Further qualified by post-hoc comparisons (see [4] in the bottom panel).
		Separate ANOVA for Painful Prosody: [1] At FC: Facial expression *p* = 0.012, *η*_*p*_^2^ = 0.335. [1] At FC: Facial expression x Intelligibility *p* = 0.006, *η*_*p*_^2 = ^0.389. **Painful faces elicited larger P2 than neutral faces when preceded by utterances with a painful prosody in a fictional language**. [2] At CP: Intelligibility *p* = 0.036, *η*_*p*_^2^ = 0.335. **Faces that were preceded by intelligible utterances with painful prosody elicited larger P2 than faces preceded by utterances in a fictional language with painful prosody**. Separate ANOVA for Neutral Prosody n.s. (*p* > 0.1).	[3] Planned comparisons: **Neutral facial expressions preceded by painful prosody elicited more negative N2−N3 (reduced empathic response) when compared to neutral faces preceded by neutral prosody**, *p* = 0.008. **Painful facial expressions preceded by painful prosody elicited more positive N2–N3 (larger empathic reaction) when compared to neutral faces preceded by neutral prosody**, *p* = 0.002. **Painful facial expressions preceded by neutral prosody did elicit an N2−N3 empathic reaction relative to neutral faces preceded by neutral prosody**, *p* = 0.017.	[3] Planned comparisons: Painful facial expressions elicited larger P3 than neutral facial expressions at **right FC**, *p* = 0.033, but not at left FC, *p* > 0.05.[4] Planned comparisons: **Intelligible utterances pronounced with painful prosody elicited larger P3 than those pronounced with neutral prosody**, *p* = 0.043. **P3 for unintelligible utterances pronounced with painful prosody decreased relative to neutral prosody**, *p* = 0.021.

#### Correlational analysis results

The *perceptual cue reaction* on the P2 component was qualified as an empathic reaction associated with affective empathy, i.e. experience-sharing, as it significantly correlated with the affective subscale of the IRI, the empathic concern (EC) at *CP*, *r* = 0.516, *p* = 0.017 (the correlation was not significant at *FC*, *r* = 0.326, *p* = 0.101) but not with EQ, max *r* = −0.116, min *p* = 0.328. The same reaction marginally correlated with EC on the N2–N3 at *CP*, *r* = 0.390, *p* = 0.061 (at *FC*, *r* = 0.315, *p* = 0.109), but did not correlate with the EQ, max *r* = 0.099, min *p* = 0.352.

**Figure 5:**
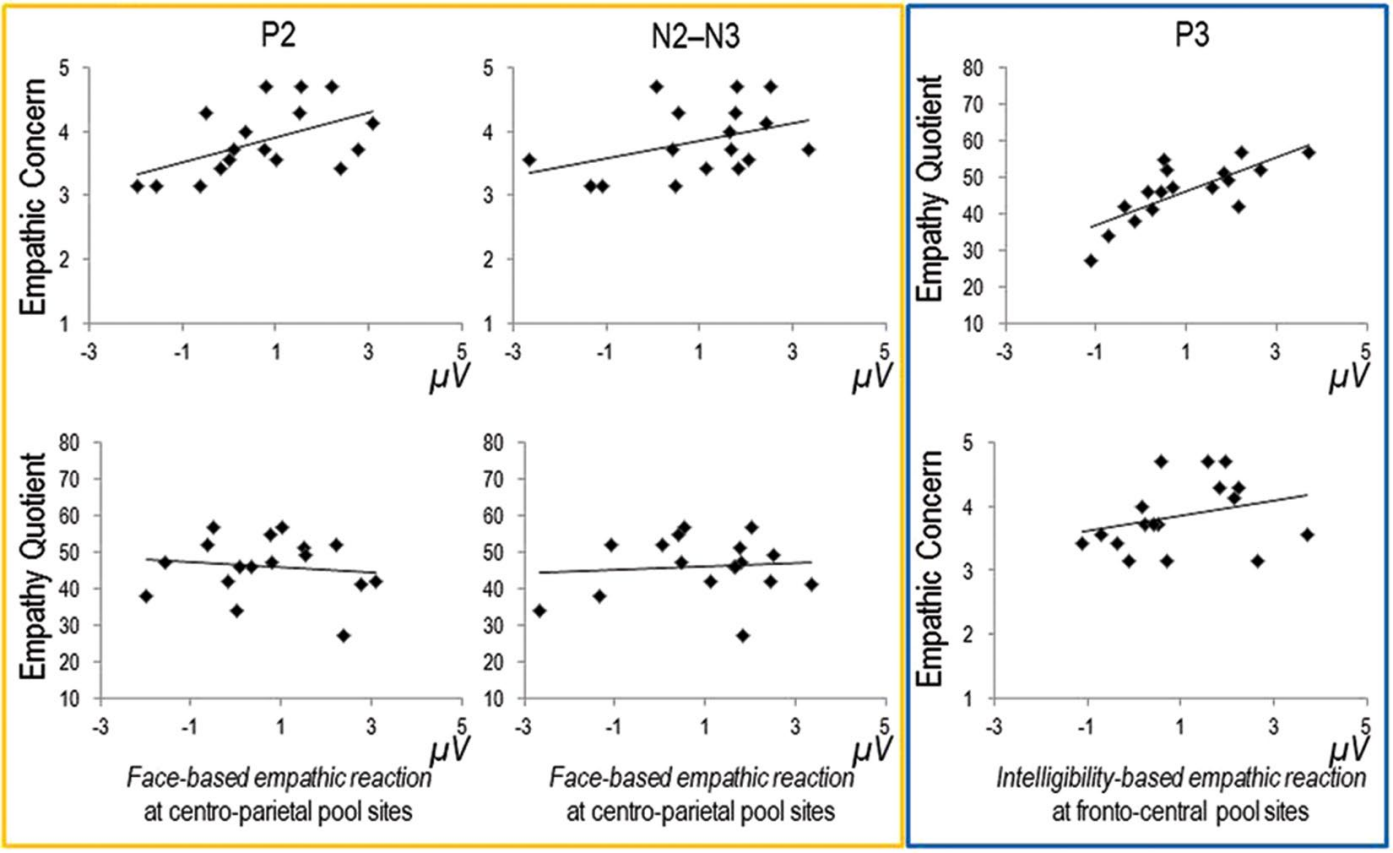
Scatter plots of the correlations between ERP empathic reactions and self-report measures of dispositional empathy.

The *semantic cue reaction* on the P3 component, positively correlated with the EQ (*r* = 0.517–0.751, max *p* = 0.017), but not with the EC, max *r* = 0.290, min *p* = 0.129, associating this empathic reaction with cognitive empathy, i.e. mentalizing, see Fig. 5.

## Discussion

In the current study, we investigated the role of affective prosody in neural empathic responses to physical pain expressed by pre-verbal and verbal cues of pain, (i.e. facial expressions and utterances). We orthogonally manipulated facial expressions (neutral vs. painful), intelligibility of the utterances (intelligible vs. unintelligible, i.e. utterances in mother-tongue vs. utterances in fictional language) and affective prosody (neutral vs. painful). On each trial of the experimental design a face stimulus was presented at the centre of the computer screen, either with a neutral or a painful expression; it was preceded at variable intervals by an utterance, either intelligible or unintelligible, pronounced with either a neutral prosody or an affective prosody expressing the speaker’s pain. All ERPs waveforms were time-locked to the presentation of the face stimuli. Importantly, intelligible utterances (i.e., utterances in mother-tongue) always considered a painful content. Our purpose was to monitor ERP empathic responses to others’ pain time-locked to the onset of faces (manifested as a positive shift of ERPs reflecting painful when compared to neutral conditions) as a function of all the combinations of cues of pain. We were interested in replicating our previous findings^16^, in which we demonstrated that when time-locked to faces onset, P2 and N2–N3 empathic responses to others’ pain were driven by facial expressions, whereas P3 empathic responses were driven by higher level cues of pain such as the painful content (i.e., choice of words) of a verbal expression. These two different temporal-windows are functionally dissociable and very likely reflections of experience-sharing and mentalizing components of empathy as supported also by source analysis^7,16,21^. Most important, the main aim of the present investigation was to elucidate how affective prosody influenced early and late empathic responses to pain. We hypothesised that because of its dual-nature – pre-verbal but also accompanying the semantic content of the speech – prosody could interact with facial expression within an early temporal window of processing and with semantic content within a later temporal window of processing.

Replicating our previous study^16^, we time-locked ERP analysis to the onset of faces and observed that painful facial expressions modulated P2 and N2–N3 components associated with the experience-sharing response. Painful contexts maximally triggered the P3 response linked to mentalizing, as further corroborated by participants’ self-rated empathy and by the pattern of correlational analysis.

Crucially, we observed that painful prosody acted on a pre-verbal domain enhancing ERP empathic reaction to painful faces when preceded by unintelligible utterances, i.e. in the fictional language, within the time-window associated with experience-sharing, including the P2 component. Painful prosody acted on a verbal domain enhancing P3 empathic reaction to painful semantic content, linked to mentalizing mechanisms. This effect of empathic reaction enhancement to painful facial expressions due to painful prosodic information was absent within the N2–N3 temporal window. N2–N3 amplitude to neutral facial expressions preceded by utterances with painful prosody was significantly less positive than that elicited by neutral facial expression preceded by utterances with neutral prosody. This pattern was opposite to what is usually observed in ERP studies on empathy. This may suggest that the incongruence between prosody and facial expression interfered with the elicitation of an empathic response. Nevertheless, this further observation strongly corroborates the view that prosody and facial expression information may interact within this earlier temporal window, including the P2 and N2–N3. Notably, a similar interference in the elicitation of neural empathic response was observed on the P3 component under conditions in which unintelligible utterances where pronounced with a painful prosody. This finding is particularly interesting when contrasted with the empathic response enhancement that we observed for intelligible utterances pronounced with a painful prosody. This pattern seems to suggest that prosodic information may magnify a higher-level empathic response linked to language (and to mentalizing) only when it is associated with a semantic content.

This pattern of neural responses translated into higher scores of self-rated empathy under conditions in which utterance were pronounced with a painful compared to a neutral prosody along with higher scores recorded when both facial expressions and prosody were painful and for intelligible, relative to unintelligible, utterances reported with painful prosody when compared to other combinations.

Taken together, these findings are consistent with those studies on on-line processing of prosodic information showing that vocal emotion recognition, i.e. prosody, can occur pre-attentively and automatically in the time-range including the Mismatch Negativity (MMN^38^) and the P2^39,40^. The MMN has been shown to peak at about 200 ms in an oddball task where standard and deviant stimuli were emotionally and neutrally spoken syllables. The differential MMN response to such comparison, larger for emotional than neutral stimuli, could therefore be taken as an index of the human ability to automatically derive emotional significance from auditory information even when irrelevant to the task. The modulations of the P2 have been related to the salience of the stimulus that conveys emotional content^39^. Importantly, the modulations of the centro-parietal P2 can also reflect the processing of the information important in a specific context: P2 is also modulated by individual characteristics of participants and experimentally-induced knowledge about categories of visual stimuli that are physically equivalent in the context of empathy for pain^23^. In line with Schirmer and Kotz^40^, evaluation of prosody encompasses a later verbal stage of processing that is related to the context evaluation and semantic integration with earlier pre-verbal bottom-up prosodic cues. When participants are required to detect an emotional change from vocal cues that can convey either prosodic and semantic information, ERP studies showed that such emotional change detection is reflected on larger P3^41,42^ when compared to non-violations conditions. Findings in the context of emotional change detection with high ecological validity^41^ can also help explain late modulations of the P3 as a function of bottom-up processes such as processing of facial expression observed in the present study. Although the present investigation considered neural responses time-locked to faces onset as a function of facial expression, accessibility to semantic content of pain (i.e., intelligibility) and prosody, we propose that on-line processing of prosodic information (as in the studies described above) and off-line processing of prosodic information (as in our study) could induce very similar ERP modulations encompassing temporal-windows linked to pre-verbal and verbal domains.

Interestingly, affective prosody also showed interactive effects with intelligibility of the utterances in a very early time-window, i.e. on the P2 (i.e., neutral faces preceded by utterances in mother-tongue with painful prosody induced a larger P2 reaction when compared to neutral faces preceded by utterances in a fictional language with painful prosody), and with the facial expression in the latest time-window, i.e. on the P3, confined to the right hemisphere at the centro-parietal sites (i.e., painful facial expressions elicited larger P3 than neutral facial expressions when preceded by utterances with painful prosody independently of their intelligibility). Within this framework, affective prosody of pain has a distinct role in enhancing neural empathic reactions by favouring the processing of congruent facial expressions of pain beyond the time-window linked to experience-sharing and favouring mentalizing processes on those faces; and, on the other side, by favouring earlier empathic reactions linked to experience-sharing to those neutral facial expressions that were preceded by utterances with a content of pain (i.e., intelligible utterances).

Importantly, similar to our previous work^16^, we did not find evidence of an interaction between facial expression and intelligibility within the earlier and the later time-windows. Remarkably, despite the higher ecological validity of the present stimuli when compared to our previous work where facial expressions were preceded by written sentences in third person (e.g., “This person got their finger hammered”), facial expression and intelligibility never interacted within both the earlier and the later time-windows, indexing that pre-verbal and verbal domains of processing distinctively contribute to the occurrence of the empathic response.

This whole pattern of results dovetails nicely with the ascertained view that affective prosody processing is a phylogenetically and ontogenetically ancient pre-verbal ability that develops along with intelligibility abilities. Similarly, it has been suggested that affective and cognitive components of empathy, i.e. experience-sharing and mentalizing, might have evolved along two different evolutionary trajectories attributing phylogenetically older age to experience-sharing than to mentalizing^43–45^. Explicit inference on others’ inner states is believed to be a higher-order cognitive ability that is shared only by apes and humans^46,47^ and its selection might be associated with increasing of social interactions complexity due to groups exchanges^48^.

## Conclusions

In the present study we provided evidence that affective prosody is a powerful communication signal of others’ pain by virtue of its dual-nature that conserved its evolutionary value along with human cognitive development. It enhances young adult humans’ explicit ability to share others’ pain acting transversely on empathy systems in two successive temporal windows. From a broader perspective, these findings may explain how harmonic interactions may survive partial or degraded information (i.e., when the speaker’s words are not understandable or their facial expression is not visible) and allow powerful communication in contexts of immediate necessity, for instance, as in case of others’ physical injuries.

## Methods

### Participants

Prior to data collection, we aimed to include 15–20 participants in the ERP analyses because it is suggested to be an appropriate sample in this field^15,19^. Data were collected from twenty-seven volunteers (10 males) from the University of Padova. Data from ten participants were discarded from analyses due to excessive electrophysiological artifacts, resulting in a final sample of seventeen participants (5 males; mean age: 24.29 years, SD = 3.72; three left-handed). By using G*Power 3.1^49^ for a 3 × 2 × 2 × 2 × 2 × 2 repeated measures design, we calculated that for 95% of power given the smallest effect size we observed, 14 was an adequate sample size. Analyses were conducted only after data collection was complete. All participants reported normal or corrected-to-normal vision, normal hearing and no history of neurological disorders. Written informed consent was obtained from all participants. The experiment was performed in accordance with relevant guidelines and regulations and the protocol was approved by the Ethical Committee of University of Padova.

### Stimuli

Stimuli were sixteen Caucasian male faces, with either a neutral or painful expression^19^ as the perceptual cue (*pre-verbal domain*) and sixteen utterances, with either unintelligible or intelligible emotional content as the semantic cue (*verbal domain*). The face stimuli were scaled using an image-processing software to fit in 2.9° × 3.6° (width x height) rectangle from a viewing distance of approximately 70 cm.

The sentences were uttered by a professional Italian actor and presented by a central speaker at an average value of 52.5 dB. Eight utterances were in participants’ mother-tongue (i.e., Italian) and each of them described a painful situation reported in first-person. Eight utterances were unintelligible (i.e., fictional language). Critically, each sentence was uttered with both neutral and painful prosody (i.e., prosodic cue). The Italian utterances were comparable for syntactic complexity, i.e., noun + verbal phrase (e.g., “I hurt myself with a knife”). The utterances in a fictional language were paired to Italian utterances for length and prosody.

To confirm that intelligibility did not affect prosody and vice versa, we tested 20 subjects for a rating task. In two separate blocks, subjects were asked to report (within a 7 points Likert scale) the pain intensity and how much the utterances were conceptually understandable (counterbalanced). We found that there was no significant difference in the pain rating with regard to the prosody (i.e., the tone of the voice) between intelligible and unintelligible utterances (*t* = 1.59, *p* = 0.11). Further, there was no significant difference in the intelligibility of the sentences between painful and non-painful prosody (*t* = −1.01, *p* = 0.31). Finally, we tested whether the painful prosody was actually perceived more intense than the neutral one, finding a significant difference (*t* = −54.38, *p* < 0.001).

Participants were exposed to an orthogonal combination of the 16 faces, and the 16 sentences uttered with both neutral and painful prosody. Stimuli were presented using E-prime on a 17-in cathode ray tube monitor with 600 × 800 of resolution and 75 Hz of refreshing rate.

### Experimental design

**Figure 6:**
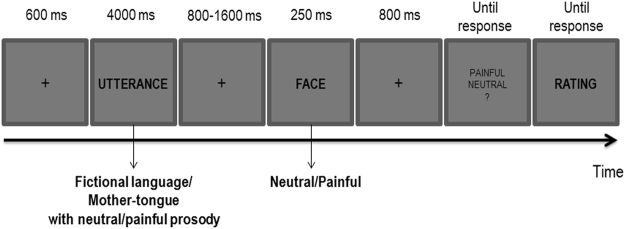
Experimental Procedure.

We implemented a variant of the pain decision task^19^. Each trial began with a central fixation cross (600 ms), followed by the utterances (i.e., semantic and prosodic cues; 4000 ms). After a blank interval (800–1600 ms, jittered in steps of 100 ms), the face (i.e., perceptual cue) was displayed for 250 ms (Fig. 6).

Participants were told that in each trial they would have heard a voice reporting potential important information to understand what the person displayed immediately after was feeling. Their task was to decide whether the face had a neutral or a painful expression by pressing one of two counterbalanced response keys. At the end of each trial, they were required to self-rate their empathy on a 7-points Likert scale for each face considering the preceding utterance. Following a brief session of practice, participants performed 320 trials in 5 blocks where all conditions were randomly intermixed. EEG was recorded while executing the pain decision task. At the end of the recording session, participants were administered with self-report questionnaires of dispositional empathy: The Italian version of the Empathy Quotient (EQ^25,50^) and the Italian version of the Interpersonal Reactivity Index (IRI^22^). The EQ has been mainly linked to cognitive aspects of empathy. The IRI is composed of four subscales measuring both affective and cognitive aspects of empathy: empathic concern, EC, and personal distress, PD; perspective taking, PT, and fantasy, FS, respectively).

### Electrophysiological recording and analyses

The EEG was recorded from 64 active electrodes placed on an elastic Acti-Cap according to the 10/20 international system, referenced to the left earlobe. The EEG was re-referenced offline to the average of the left and right earlobes. Horizontal EOG was recorded bipolarly from two external electrodes positioned laterally to the external canthi. Vertical EOG was recorded from Fp1 and one external electrode placed below the left eye. The electrode impedance was kept less than 10 KΩ. EEG and EOG signals were amplified and digitized at a sampling rate of 250 Hz (pass band 0.01–80 Hz). The EEG was segmented into 1200-ms epochs starting 200 ms prior to the onset of the faces. The epochs were baseline-corrected based on the mean activity during the 200-ms pre-stimulus period. Trials associated with incorrect responses or contaminated by horizontal and vertical eye movements or other artifacts (exceeding ± 60μV and ± 80μV, respectively) were discarded from analysis. We kept participants who showed at least 20 trials in each condition. The final range of trials was 21–40 but only 3 participants showed less than 25 trials in at least one condition. Separate average waveforms for each condition were then generated time-locked to the presentation of the faces as a function of the preceding utterances. Statistical analyses of ERPs mean amplitudes focused on P2 (125–170 ms), N2–N3 (180–380 ms) and P3 (400–900 ms). The selection of a single temporal window including the N2 and N3 components was mainly based on our previous studies^16,21^ because it was critical for the purpose of the present investigation to replicate our previous findings on the dissociable nature of empathic responses triggered by facial expressions and other higher-level cues of pain. Mean ERP amplitude values were measured at four pooled sites from right fronto-central (rFC: F2, F4, F6, FC2, FC4, FC6) and centro-parietal (rCP: CP2, CP4, CP6, P2, P4, P6) regions, and from left fronto-central (lFC: F1, F3, F5, FC1, FC3, FC5) and centro-parietal (lCP: CP1, CP3, CP5, P1, P3, P5) regions.

### Statistical analysis

#### Pain Decision Task

Reaction times (i.e., RTs) exceeding each individual mean RT in a given condition +/− 2.5 SD and associated with incorrect responses were excluded from analyses. RTs and mean proportions of correct responses were submitted to a repeated measure ANOVA including facial expression (neutral vs. painful), intelligibility (mother-tongue, i.e., Italian vs. fictional language) and prosody (neutral vs. painful) as within-subjects factors. ANOVAs carried out on mean amplitude values of each ERP component also included the within-subjects factor hemisphere (right vs. left) and were carried out separately for *FC* and *CP*.

The significant threshold for all statistical analyses was set to 0.05. Exact *p* values, mean squared errors (i.e., *MS*_*e*_) and effect sizes (i.e., partial eta-squared, *η*_*p*_^2^) are reported. Confidence intervals (i.e., CIs, set at 95% in squared brackets) are defined only for paired t-tests and referred to difference of means (i.e., *M*_*diff*_). Planned comparisons relevant to test the hypotheses of the present experiment are reported. Bonferroni correction was applied for multiple comparisons.

#### Correlational analysis

With the aim of further qualifying neural responses as experience-sharing or mentalizing responses we correlated ERP empathic reactions (i.e., painful minus neutral conditions) with participants’ dispositional empathy as measured by the IRI and the EQ. More specifically, the painful-minus-neutral score was computed for both the pre-verbal and verbal domains of processing. A *perceptual cue reaction* was computed for the pre-verbal domain by subtracting ERP to neutral faces preceded by utterances with neutral prosody from ERP to painful faces preceded by utterances with neutral prosody regardless of the intelligibility and of the hemisphere. A *semantic cue reaction* was computed for the verbal domain by subtracting ERP to faces as a function of utterances in a fictional language from ERP to faces as a function of Italian utterances regardless of facial expression, prosody and hemisphere. For both reactions, positive values indexed an empathic reaction.

## Data Availability

The datasets generated during and/or analysed during the current study are not publicly available because we did not obtain from participant consent for publication but are available from the corresponding author on reasonable request.
