# Publisher Correction: Neural measures of the role of affective prosody in empathy for pain

**DOI:** 10.1038/s41598-019-40292-4

**Published:** 2019-03-15

**Authors:** Federica Meconi, Mattia Doro, Arianna Schiano Lomoriello, Giulia Mastrella, Paola Sessa

**Affiliations:** Department of Developmental and Social Psychology, University of Padova, Padova, Italy

Correction to: *Scientific Reports* 10.1038/s41598-017-18552-y, published online 10 January 2018

In the original version of this Article, the author Arianna Schiano Lomoriello was incorrectly indexed. This error has now been corrected.

